# Are perceived neighbourhood problems associated with the likelihood of smoking?

**DOI:** 10.1136/jech.2007.068767

**Published:** 2008-12-05

**Authors:** A Ellaway, S Macintyre

**Affiliations:** MRC Social & Public Health Sciences Unit, Glasgow, UK

## Abstract

**Objective:**

To explore associations between residents’ perceptions of the local residential environment and the likelihood of their smoking.

**Design:**

Using data (n = 2615) from the West of Scotland Twenty-07 Study, separately by gender, cross-sectional associations between respondents’ perceptions of neighbourhood (perceived absence of goods, incivilities and physical environmental problems) and the likelihood of being a current smoker and the amount smoked were examined.

**Results:**

Perceived neighbourhood problems are associated with the likelihood of smoking but mainly among those with the most negative view of the local neighbourhood. Perceptions of the provision of neighbourhood amenities seems to be more strongly associated with women’s than men’s smoking status, whereas the perceived quality of the local neighbourhood appears to be a better predictor of men’s smoking.

**Conclusions:**

Efforts to reduce smoking levels among more deprived groups may need to pay more attention to the role of local environmental conditions in influencing smoking behaviour.

A number of studies have shown that where people live is associated with the likelihood of smoking, even after controlling for individual socioeconomic factors.^1^^–^10 Various explanations for this observation include local norms and culture,^11^ ^12^ and smoking as a potential coping mechanism to mediate stresses, including those associated with living in an unpleasant or threatening residential environment.^13^ ^14^

The degree to which people perceive their residential environment to be pleasant or otherwise has been shown to be associated with various health outcomes.^15^^–^20 We explore associations between residents' perceptions of the local residential environment and the likelihood of smoking. Few studies have examined this issue, with most previously published work relying on a fairly narrow range of neighbourhood perceptions.^17^ ^21^ ^22^ Our data allow us to examine several dimensions of perceived neighbourhood conditions. We do this separately for men and women, as gender differences have been shown in the associations between health and the experience of place^23^^–^25 and the determinants of smoking.^26^^–^28

## METHODS

### Study population

The analysis used a subset of data from the fourth sweep (conducted in 2001) of the West of Scotland Twenty-07 Study: Health in the Community.^29^ At this sweep 2661 respondents took part, 2615 of whom had complete data for the variables examined here. The survey respondents are broadly representative of the West of Scotland population.

### Sociodemographic measures

The survey involves three cohorts: the mean age of each cohort when interviewed was 30 (n = 824), 50 (n = 967) and 68 (n = 824) year respectively; 1437 women and 1178 men.

Registrar General’s occupation-based classification scheme^30^ using the last known occupation of the head of the respondent’s household was used to determine social class.

### Perceived neighbourhood problems

Respondents were asked about 16 types of socioenvironmental problems in their neighbourhood, and invited to reply using a three-point scale (“not a problem” score 1, “minor problem” score 2, “serious problem” score 3). Using factor analysis, three domains emerged from the 16 items: “incivilities” (litter, vandalism, burglaries, disturbance by children or youths, nuisance from dogs, assaults, discarded needles/syringes, people, reputation of the neighbourhood), “absence of goods” (lack of safe play areas for children, lack of recreation facilities, difficulties obtaining services) and “physical environmental problems” (speeding traffic, derelict/waste ground, uneven/dangerous pavements, smells and fumes). For each of these three domains, a score was constructed by summing responses to each item making up the domains. These scores were subsequently divided into quartiles, with those in the lowest quartile being the most positive and those in the highest quartile being the most negative about their local neighbourhood.

### Smoking status

Respondents were asked about their smoking status, choosing from three categories: an ex-smoker, never smoked or current smoker. Under a third (30.6% men and 28% women) in our sample were current smokers (n = 763), which is broadly similar to the Scottish population as a whole.^31^ Current smokers were asked about the number of cigarettes or cigars smoked per day; this ranged from 1 to 60 per day (mean 14.24, (SD) 9.6) among men, and 1–80 per day (mean 15.07 (SD) 9.2) among women.

### Analyses

Statistical analyses were performed using SPSS V.12.0, undertaken separately for men and women, as interactions by gender were found for perceived absence of goods (p<0.001, CI 1.006 to 1.088); perceived incivilities (p<0.04, CI 1.002 to 1.090) and physical environmental problems (p<0.052, CI 1.000 to 1.081). Logistic regression was used to calculate the odds ratio (95% CI) for the likelihood of being a current smoker (as a binary variable: current smokers compared with non-current (former and never)), with age, social class and perceived neighbourhood problems entered simultaneously (separate models were constructed for absence of goods, incivilities and physical problems) with the lowest quartile (the group most positive about their local neighbourhood) being the reference category. The p value <0.05 was used as the cut-off for statistical significance.

Differences in the reported number of cigarettes smoked per day were also examined, using general liner modelling and means (adjusted for cohort and social class) were calculated.

## RESULTS

For each of our three domains of perceived neighbourhood problems, the likelihood of being a current smoker increased with rising levels of perceived problems (with the exception of perceived physical environmental problems and likelihood of smoking among women) (table 1). However, this was statistically significant only when comparing those with the highest quartiles of perceived problems with the lowest quartile. Gender differences were observed in the strength of those relationships, with perceived absence of goods being more important for women and perceived incivilities and physical environmental problems being more important for men.

**Table 1:** Odds ratio (95% CI) for likelihood of being a current smoker*

	Men		Women	
	OR (95% CI)	p Value	OR (95% CI)	p Value
*Absence of goods*				
1 Lowest score†	1		1	
2	0.79 (0.55 to 1.15)	0.219	0.91 (0.65 to 1.27)	0.583
3	1.13 (0.79 to 1.61)	0.509	1.24 (0.88 to 1.72)	0.217
4 Highest score	1.51 (1.06 to 2.14)	0.021	1.64 (1.18 to 2.28)	0.003
*Incivilities*				
1 Lowest score†	1		1	
2	1.23 (0.88 to 1.73)	0.233	1.02 (0.75 to 1.39)	0.884
3	1.27 (0.87 to 1.84)	0.213	1.18 (0.84 to 1.65)	0.332
4 Highest score	2.21 (1.53 to 3.20)	0.001	1.42 (1.01 to 2.03)	0.045
*Physical environmental problems*				
1 Lowest score†	1		1	
2	1.06 (0.75 to 1.48)	0.755	1.11 (0.80 to 1.52)	0.536
3	1.14 (0.78 to 1.65)	0.498	1.04 (0.74 to 1.48)	0.807
4 Highest score	1.78 (1.25 to 2.53)	0.001	1.55 (1.12 to 2.15)	0.008

*Regression models based on age, social class and perceived neighbourhood problems entered simultaneously (separate models for absence of goods, incivilities and physical environmental problems).

†Reference category.

Only one domain, absence of goods, was associated with the number of cigarettes/cigars smoked in both men and women (table 2), although among men the amount smoked was similar for those with the most negative and the most positive views. Among women, those with the most negative view smoked, on average, more than three cigarettes per day more than those female smokers with the most positive view.

**Table 2:** Mean number* of cigarettes/cigars smoked per day†

	Men		Women	
	Mean	p Value‡ (F)	Mean	p Value‡ (F)
*Absence of goods*		0.024 (F = 3.18)		0.030 (F = 3.02)
1 Lowest score	14.48 (1.01)		12.38 (0.93)	
2	10.68 (1.32)		12.27 (1.14)	
3	12.08 (1.19)		14.59 (1.02)	
4 Highest score	14.67 (1.07)		15.54 (0.93)	
*Incivilities*		0.67 (F = 0.52)		0.47 (F = 0.85)
1 Lowest score	14.29 (1.12)		12.92 (0.93)	
2	12.59 (1.10)		14.36 (0.94)	
3	13.85 (1.30)		13.35 (1.07)	
4 Highest score	13.29 (1.08)		14.82 (1.10)	
*Physical environmental problems*		0.32 (F = 1.18)		0.35 (F = 1.10)
1 Lowest score	14.53 (1.04)		13.68 (0.92)	
2	11.98 (1.14)		12.88 (1.01)	
3	13.09 (1.28)		13.21 (1.18)	
4 Highest score	13.74 (1.11)		15.11 (0.96)	

*Adjusted for cohort and social class.

†Current smokers only.

‡p Values related to model as a whole.

## DISCUSSION

We have found that perceived neighbourhoods problems are associated with the likelihood of smoking but mainly among those with the most negative view of the local neighbourhood. There are several potential explanations for our findings; for example, it may be that areas which have the poorest environments are particularly influential in inducing smoking behaviour as a response to the stress of living there; it may be that social norms with regard to smoking are operating more strongly in these neighbourhoods; or it may be that cigarettes are more readily available in the poorest neighbourhoods either through more shops selling tobacco products or that contraband products are more easily and cheaply available.^32^ Because these are cross-sectional data, we cannot rule out the possibility that both smoking and negative views about the environment are influenced by a fourth factor (personality, mental health or mood), or that smokers are selected in some way into less attractive neighbourhoods.

Perceptions of the availability of neighbourhood amenities seems to be more strongly associated with women’s than men’s smoking status, whereas the quality of the local neighbourhood appears a better predictor of men’s smoking. It may be as women tend to be the main carers for children^24^ ^33^ they are more sensitised to particular aspects of their local neighbourhood, such as the availability of safe places to play. The gendered experience and consequences of place for health have only begun to be noted in a few other studies,^21^ ^23^ ^24^ suggesting a need for further research.

In conclusion, smoking is a major public health concern and a major contributor to socioeconomic inequalities in health.^34^ ^35^ People who smoke in deprived areas are less likely to give up smoking than their more affluent counterparts^36^ and efforts which target individuals have had a limited effect among more deprived groups.^37^^–^39 Our results suggest that closer attention may need to be paid to the role of local environmental conditions in influencing smoking behaviour.

**What is already known on this subject**

- A number of studies have shown that area of residence is associated with the likelihood of smoking, although the precise mechanisms are not well understood as yet.
- Some recent studies have found that perceptions of the local environment (as pleasant or otherwise) may contribute to smoking behaviour.

**What this study adds**

- This study found that residents with very negative views of their local neighbourhood are more likely to smoke, even after taking individual characteristics into account.
- Women’s smoking status is associated with a perceived lack of local amenities (eg safe play areas for children), whereas the quality of the local neighbourhood (eg presence of litter and graffiti) appears to be a better predictor of men’s smoking.

**Policy implication**

Closer attention may need to be paid to the role of local environmental conditions in potentially influencing smoking behaviour.
